# Bone marrow‐derived mesenchymal stem cells promote *Helicobacter pylori*‐associated gastric cancer progression by secreting thrombospondin‐2

**DOI:** 10.1111/cpr.13114

**Published:** 2021-08-25

**Authors:** Huiying Shi, Cuihua Qi, Lingjun Meng, Hailing Yao, Chen Jiang, Mengke Fan, Qin Zhang, Xiaohua Hou, Rong Lin

**Affiliations:** ^1^ Department of Gastroenterology Union Hospital Tongji Medical College Huazhong University of Science and Technology Wuhan China; ^2^ Department of Pathology Union Hospital Tongji Medical College Huazhong University of Science and Technology Wuhan China

**Keywords:** Bone marrow‐derived mesenchymal stem cells (BM‐MSCs), Cancer‐associated fibroblasts (CAFs), Gastric cancer (GC), *Helicobacter pylori*, Thrombospondin‐2

## Abstract

**Objectives:**

Bone marrow‐derived cells (BMDCs), especially mesenchymal stem cells (MSCs), may be involved in the development of *Helicobacter pylori*‐associated gastric cancer (GC) in mice, but the specific mechanism remains unclear, and evidence from human studies is lacking.

**Materials and Methods:**

To verify the role of BM‐MSCs in *H pylori*‐associated GC, green fluorescent protein (GFP)‐labelled BM‐MSCs were transplanted into the subserosal layers of the stomach in a mouse model of chronic *H pylori* infection. Three months post‐transplantation, the mice were sacrificed, and the gastric tissues were subjected to histopathological and immunofluorescence analyses. In addition, we performed fluorescence in situ hybridization (FISH) and immunofluorescence analyses of gastric tissue from a female patient with *H pylori* infection and a history of acute myeloid leukaemia who received a BM transplant from a male donor.

**Results:**

In mice with chronic *H pylori* infection, GFP‐labelled BM‐MSCs migrated from the serous layer to the mucosal layer and promoted GC progression. The BM‐MSCs differentiated into pan‐cytokeratin‐positive epithelial cells and α‐smooth muscle actin‐positive cancer‐associated fibroblasts (CAFs) by secreting the protein thrombospondin‐2. FISH analysis of gastric tissue from the female patient revealed Y‐chromosome‐positive cells. Immunofluorescence analyses further confirmed that Y‐chromosome‐positive cells showed positive BM‐MSCs marker. These results suggested that allogeneic BMDCs, including BM‐MSCs, can migrate to the stomach under chronic *H pylori* infection.

**Conclusions:**

Taken together, these findings imply that BM‐MSCs participate in the development of chronic *H pylori*‐associated GC by differentiating into both gastric epithelial cells and CAFs.

## Graphical abstract

Bone marrow mesenchymal stem cells transplanted in mice with chronic *H pylori* infection gradually migrate from the subserosa to the mucosa and secret THBS2 protein to promote the occurrence and development of gastric cancer.

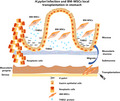

## 1 INTRODUCTION

Gastric cancer (GC) is one of the most common malignant tumours worldwide. According to the latest global cancer data released in 2020, GC ranks 6th in incidence and 4th in mortality among all malignancies.^1^ Risk factors for GC include chronic *Helicobacter pylori* infection, alcohol consumption,^2^, ^3^ tobacco smoking^4^, ^5^ and consumption of foods preserved by salting,^6^, ^7^ among others. Chronic *H pylori* infection is considered the principal cause of non‐cardia GC^1^; approximately 90% of new cases of non‐cardia GC are associated with *H pylori* infection.^8^, ^9^ Nearly half of the global population is infected with *H pylori*, making it the most common infection in the world.^10^, ^11^ Chronic *H pylori* infection is thought to cause gastric epithelial hyperplasia and mitotic error, leading to metaplasia, dysplasia and ultimately adenocarcinoma.^12^ However, beyond these histological characteristics, the mechanisms by which *H pylori*‐associated GC originates and progresses are not fully understood.^13^

Previous studies suggested that GC is caused by malignant transformation of gastric mucosal epithelial cells,^14^, ^15^, ^16^ but recent cell lineage tracing studies have proposed that bone marrow‐derived cells (BMDCs) are the cell source of GC.^17^, ^18^ Bone marrow‐derived mesenchymal stem cells (BM‐MSCs) have multi‐lineage differentiation potential and play an important role in tissue repair.^19^ The tropism of BM‐MSC for sites of tissue damage and the tumour microenvironment has been confirmed,^20^, ^21^ and evidence indicates that the tissue‐regenerative function of MSCs may go awry in malignant tumours.^22^, ^23^, ^24^, ^25^, ^26^ Nonetheless, the exact role of BM‐MSCs in *H pylori‐*associated GC and the underlying mechanisms remain unclear.

BM‐MSCs have been shown to promote tumour development in various cancer types by developing into cancer‐associated fibroblasts (CAFs). CAFs play a key role in tumorigenesis by regulating the tumour microenvironment and affecting tumour cell behaviour. Upon tissue damage, epithelial cell transformation prompts the recruitment of several types of cells and their reprogramming into CAFs^27^; the most direct sources of CAFs are resident tissue fibroblasts and MSCs.^27^, ^28^, ^29^ α‐smooth muscle actin (α‐SMA) is a robust CAF marker that is commonly used to identify CAFs with myofibroblast morphology.^30^, ^31^ CAFs can also be further induced to secrete cytokines that promote tumour cell growth and invasion.^32^, ^33^, ^34^, ^35^ Multiple lines of evidence suggest that a significant proportion of CAFs in tumours originates from BM‐MSCs.^26^ For instance, in a mouse model of pancreatic ductal adenocarcinoma, BM‐MSCs are recruited into the tumour microenvironment, where they differentiate into CAFs and secrete VEGF to promote tumour progression.^36^ BM‐MSCs promote tumour growth by secreting interleukin‐6 as CAFs in a murine ovarian carcinoma xenograft model.^37^ Therefore, in this study, we aimed to investigate whether BM‐MSCs can promote the progression of *H pylori‐*associated GC as CAFs and the underlying mechanism. To do so, we transplanted BM‐MSCs into a mouse model of chronic *H pylori* infection.

## 2 MATERIALS AND METHODS

### 2.1 Establishment of BM‐MSCs transplantation in chronic *H pylori*‐infected mice

Four‐ to six‐week‐old male BALB/C mice were purchased from Beijing HFK Bioscience Co., LTD., and raised in a specific pathogen‐free (SPF) animal feeding room at the Animal Center of Tongji Medical College in a constant‐temperature (21‐25℃), constant‐humidity (50%‐60%) environment. The mice had free access to standard rodent diet and water before the experiment. All procedures were conducted strictly in accordance with the Guide for the Care and Use of Laboratory Animals, and the experiments were approved by the Laboratory Animal Ethics Committee of Huazhong University of Science and Technology.

The mice were randomly allocated to 4 groups: the phosphate‐buffered saline (PBS) transplantation group (SHAM, n = 10), BM‐MSCs transplantation group (BM‐MSCs, n = 10), chronic *H pylori* infection group (Hp, n = 20) and chronic *H pylori* infection plus BM‐MSCs transplantation group (Hp +BM‐MSCs, n = 27).

To establish the model of chronic *H pylori* infection, mice were inoculated orally with a suspension of *H pylori* strain SS1 (0.1 ml, 1‐2 × 10^9^ CFU/mL) thrice over a 5‐day period using a mouse gavage needle.^38^ The SHAM and BM‐MSCs groups were mock‐inoculated with *H pylori* liquid culture medium. Three months after successful infection with *H pylori* strain SS1, green fluorescent protein (GFP)‐labelled BM‐MSCs (2 × 10^6^ cells in 0.1 ml of PBS) were transplanted into the antrum area of the greater curvature into the subserosa. Three months after transplantation, the mice were sacrificed, stomach samples were collected, and gastric tissues were subjected to histological analysis. The distribution of GFP‐labelled BM‐MSCs in the stomach was detected by flow cytometry. Laser confocal immunofluorescence microscopy was used to observe the migration and distribution of GFP‐labelled BM‐MSCs in the stomach and to analyse the co‐expression of GFP and the CAF marker α‐smooth muscle actin (α‐SMA) or gastric epithelial cell marker pan‐cytokeratin (pan‐CK).

### 2.2 In vivo tumorigenesis in nude mice

Male BALB/c nude mice (HFK BIOSCIENCE CO., LTD, Beijing, China) were bred in a licenced SPF laboratory at the Animal Center of Tongji Medical College. To assess the effect of BM‐MSCs on tumour growth in vivo, 5‐week‐old nude mice (20 g body weight) were randomly allocated to the following groups (n = 6): 1) control mice injected with PBS (Control group); 2) mice injected with 2 × 10^6^ gastric cancer cell line MFCs (MFC group); 3) mice injected with 2 × 10^6^ BM‐MSCs (BM‐MSCs group); and 4) mice injected with 2 × 10^6^ MFCs mixed with 2 × 10^6^ BM‐MSCs (MFC +BM‐MSCs group). In all groups, cells were resuspended in 200 μl of PBS and inoculated subcutaneously into the right armpit of each mouse. After transplantation, the subcutaneous tumour was measured with Vernier callipers to calculate tumour size, and the tumour volume was calculated according to the formula (L × W^2^) × 0.5, where L is the length of each tumour and W is the width of each tumour. The mice were sacrificed 1‐3 weeks later, and the subcutaneous tumours were analysed by histological staining. All procedures were approved by the Experimental Animal Ethics Committee of Huazhong University of Science and Technology.

### 2.3 iTRAQ analysis

Isobaric tags for relative and absolute quantification (iTRAQ), a method of quantitative proteomics, were used to screen and identify proteins secreted by BM‐MSCs after *H pylori* treatment. BM‐MSCs were cultured in a dish with culture medium until reaching confluence and were then treated with or without a supernatant of *H pylori* at a multiplicity of infection (MOI) of 50 for 12 h. Conditioned and non‐conditioned medium of BM‐MSCs was harvested and centrifuged at 4000 rpm for 10 min. The non‐conditioned medium of BM‐MSCs was used as a control. Then, the samples were processed and detected by Shanghai Luming Biotechnology Co., LTD. The relative abundance of proteins was calculated based on individual peptide ratios, and the threshold for differentially expressed proteins was ≥1.5 or ≤0.75.

### 2.4 Short hairpin RNA transfection

To stably knock down thrombospondin‐2 (THBS2) in BM‐MSCs, a lentiviral vector (U6‐MCS‐Ubiquitin‐Cherry‐IRES‐puromycin) containing a short‐hairpin RNA (shRNA) for the THBS2 coding sequence (NM_011581) was constructed (Gene Technologies, Inc Shanghai, China). The cells were seeded in full growth medium in 12‐well tissue culture dishes at 10,000 cells/well and grown at 37℃ for 18‐24 h prior to lentivirus transfection with THBS2 shRNA (Gene Technologies, Inc, Shanghai, China) or scrambled shRNA (negative control) (Gene Technologies, Inc, Shanghai, China) at a MOI of 100 according to the manufacturer's instructions. The sequence of the THBS2‐specific shRNA was GCTGTAGGTTTCGACGAGTTT, and the sequence of the negative control shRNA was TTCTCCGAACGTGTCACGT. At 48‐h post‐infection, stably transfected cell lines were selected using puromycin (Sigma‐Aldrich; Merck Millipore) at a dose of 8 μg/ml for 3 days. In THBS2 knockdown experiments, THBS2 gene expression was measured by qRT‐PCR, and THBS2 protein expression was assessed by Western blot.

### 2.5 Analysis of the effect of THBS2‐deficient BM‐MSCs on mice with chronic *H pylori* infection

To evaluate the effect of THBS2‐deficient BM‐MSCs on *H pylori*‐associated GC in chronic *H pylori*‐infected mice, mice were allocated to the following groups: 1) PBS transplantation group (SHAM group, n = 10); 2) scrambled‐shRNA (negative control) BM‐MSCs (sh‐NC‐BM‐MSCs) transplantation group (sh‐NC‐BM‐MSCs group, n = 10); 3) THBS2‐specific shRNA BM‐MSCs (sh‐THBS2‐BM‐MSCs) transplantation group (sh‐THBS2‐BM‐MSCs group, n = 10); 4) chronic *H pylori* infection group (Hp group, n = 15); 5) chronic *H pylori* infection with sh‐NC‐BM‐MSCs transplantation group (Hp +sh‐NC‐BM‐MSCs, n = 25); and 6) chronic *H pylori* infection with sh‐THBS2‐BM‐MSCs transplantation group (Hp +sh‐THBS2‐BM‐MSCs group, n = 25).

### 2.6 Analysis of the effect of THBS2‐deficient BM‐MSCs on gastric cancer xenografts

To investigate the effect of THBS2‐deficient BM‐MSCs on tumour growth in vivo, mice were allocated to the following groups (n = 5): 1) PBS injection (Control group); 2) injection with 2 × 10^6^ gastric adenocarcinoma cell lines SGCs (SGCs group); 3) injection with 2 × 10^6^ sh‐NC‐BM‐MSCs (sh‐NC‐MSCs group); 4) injection with 2 × 10^6^ SGCs mixed with 2 × 10^6^ sh‐NC‐BM‐MSCs (SGC +sh‐NC‐MSCs group); 5) injection with 2 × 10^6^ sh‐THBS2‐BM‐MSCs (sh‐THBS2‐MSCs group); and 6) injection with 2 × 10^6^ SGCs mixed with 2 × 10^6^ sh‐THBS2‐BM‐MSCs (SGC +sh‐THBS2‐MSCs). The mice were sacrificed 2 weeks after injection, and the subcutaneous tumours were analysed by histological staining.

### 2.7 Gastric mucosal tissue biopsy analysis

A 41‐year‐old woman with acute myeloid leukaemia received a bone marrow transplant from a male donor. She had a history of chronic gastritis, duodenal ulcer and *H pylori* infection. One year after transplantation, endoscopic biopsies of gastric mucosal tissues were performed, and short tandem repeat sequence (STR) PCR was used to quantitatively analyse the chimerism of the gastric mucosal tissues. At the same time, we performed fluorescence in situ hybridization (FISH) analysis of the recipient's gastric mucosal sections using CEPY (orange)/CEPX (green) dual‐colour probes, and sequential sections were used to analyse the properties of the BMDCs by immunofluorescent staining of CD105 (red, a marker of BM‐MSCs) or CD45 (red, a marker of BM‐derived leukocytes).

### 2.8 Statistical analysis

SPSS 25.0 statistical software was used for data analyses. Data are expressed as the mean ±standard error or standard deviation of at least three independent experiments. One‐way ANOVA or the independent‐sample t test was used to compare differences between groups. The chi‐square or Fisher's exact test was used for categorical variables. *P* <.05 was considered statistically significant.

Information on protocols used for cell culture, characterization of BM‐MSCs, culture of *H pylori*, micro‐PET/CT imaging in vivo, qRT‐PCR, preparation of total cell extracts and western blot analysis, CFSE‐labeling assay, transwell migration assay, fluorescence immunohistochemistry, immunohistochemistry for paraffin‐embedded sections, HE staining and Giemsa staining, flow cytometric analysis, are provided in the Supporting information. All gene primer sequences are shown in Table S1.

## 3 RESULTS

### 3.1 BM‐MSCs promote the development of *H pylori*‐associated gastric cancer in vivo

**FIGURE 1:**
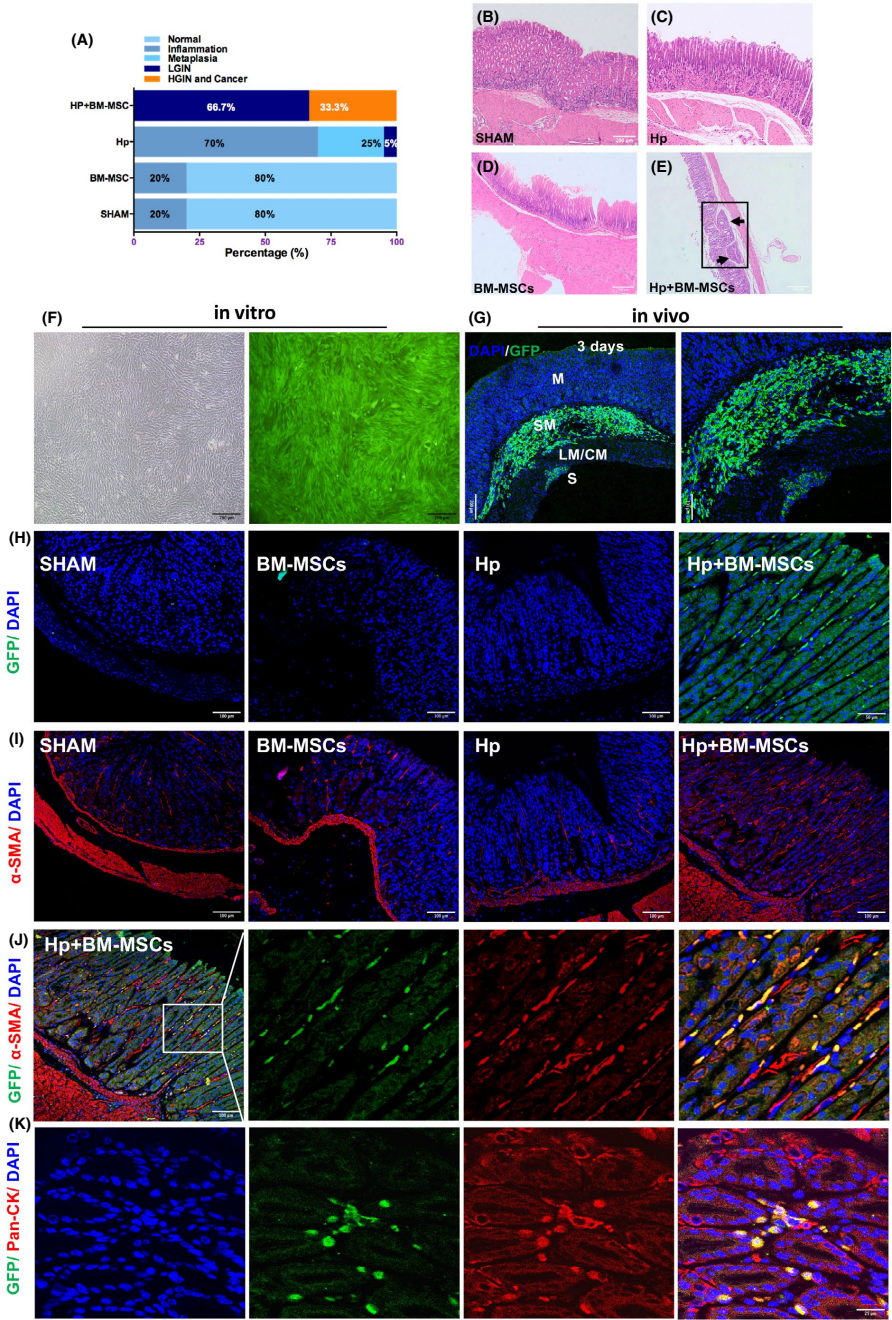
In chronic *H pylori*‐infected mice, BM‐MSCs promote *H pylori*‐associated gastric cancer progression by differentiating into α‐SMA+cells or pan‐CK+cells. A, Statistics of the histopathological analysis of each group. Representative gastric mucosa histopathology images from the B, SHAM, C, Hp, D, BM‐MSCs and E, Hp+BM‐MSCs groups are shown; the black rectangle shows that the tumour has infiltrated the submucosa. F, Representative light microscopy images (left panel) and fluorescence microscopy images (right panel) of GFP‐labelled BM‐MSCs cultured in vitro. G, The distribution of GFP‐labelled BM‐MSCs in the mouse stomach was observed by laser confocal immunofluorescence 3 days after transplantation; the transplanted cells (green) survived and migrated from the subserosa (S) to the submucosa (SM). Nuclei were labelled with DAPI (blue). H‐I, Representative immunofluorescence images of GFP^+^ cells (green) or α‐SMA^+^ cells (red) in the stomach in the SHAM, BM‐MSCs, Hp and Hp +BM‐MSCs groups, respectively. The nuclei were labelled with DAPI (blue). J‐K, Dual immunofluorescence analysis of GFP (green) and α‐SMA (red) or pan‐CK (red) expression in the stomach in the Hp +BM‐MSCs group. Nuclei were labelled with DAPI (blue). LM/CM, longitudinal muscle/circular muscle

To explore the role of BM‐MSCs in GC, BM‐MSCs were isolated (Figure S1) and locally transplanted into the stomach in mice that had been infected with *H pylori* for 3 months. At 3 months post‐transplantation, histological analysis showed that the incidence of high‐grade gastric intraepithelial neoplasia (HGIN) and GC was significantly higher in the Hp +BM‐MSCs group than in the Hp group (Figure 1A; 33.3% vs. 0%, *P* <.01). In addition, the incidence of low‐grade gastric intraepithelial neoplasia (LGIN) was significantly higher in the Hp +BM‐MSCs group (Figure 1A; 66.7% vs. 5%, *P* <.001). In the Hp group, the incidence of inflammation was 70%, the incidence of intestinal metaplasia was 25%, and the incidence of LGIN was 5%. BM‐MSCs transplantation did not promote the incidence of GC in non‐*H pylori*‐infected mice (Figure 1A; BM‐MSCs group vs. SHAM group, *P* >.05). Representative histopathological results for the SHAM group, Hp group, BM‐MSCs group and Hp +BM‐MSCs group are shown in Figure 1B‐E, and the staining images of *H pylori* in gastric tissue were showed in Figure S2A‐B. These results suggest that BM‐MSCs promote GC progression in mice with chronic *H pylori* infection.

### 3.2 BM‐MSCs transplanted in mice with chronic *H pylori* infection gradually migrate from the subserosa to the mucosa and differentiate into pan‐CK+gastric epithelial cells or α‐SMA+ CAFs.

GFP‐labelled BM‐MSCs were transplanted into the stomach in *H pylori*‐infected mice, and their distribution was tracked (Figure 1F‐G). At 3 days post‐transplantation, laser confocal immunofluorescence microscopy showed that the transplanted cells had survived and migrated from the subserosa to the submucosa (Figure 1G). At 3 months post‐transplantation, the GFP‐labelled BM‐MSCs had migrated from the serous layer to the mucosal layer in the Hp +BM‐MSCs group (Figure 1H), but no obvious migration was detected in the BM‐MSCs group by laser confocal immunofluorescence microscopy (Figure 1H) or flow cytometry (Figure S2C).

To further elucidate the specific role of BM‐MSCs in GC pathogenesis, we analysed the co‐localization of GFP‐labelled BM‐MSCs with expression of the CAF marker α‐SMA or gastric epithelial cell marker pan‐CK (Figure 1I‐K). Laser confocal immunofluorescence analysis revealed co‐localization of GFP and α‐SMA (red) expression (Figure 1J) as well as GFP and pan‐CK (red) expression (Figure 1K) in the Hp +BM‐MSCs group. These results indicate that BM‐MSCs can differentiate into pan‐CK+gastric epithelial cells and α‐SMA+CAFs.

### 3.3 *H pylori* enhances the proliferation and migration of BM‐MSCs in vitro

**FIGURE 2:**
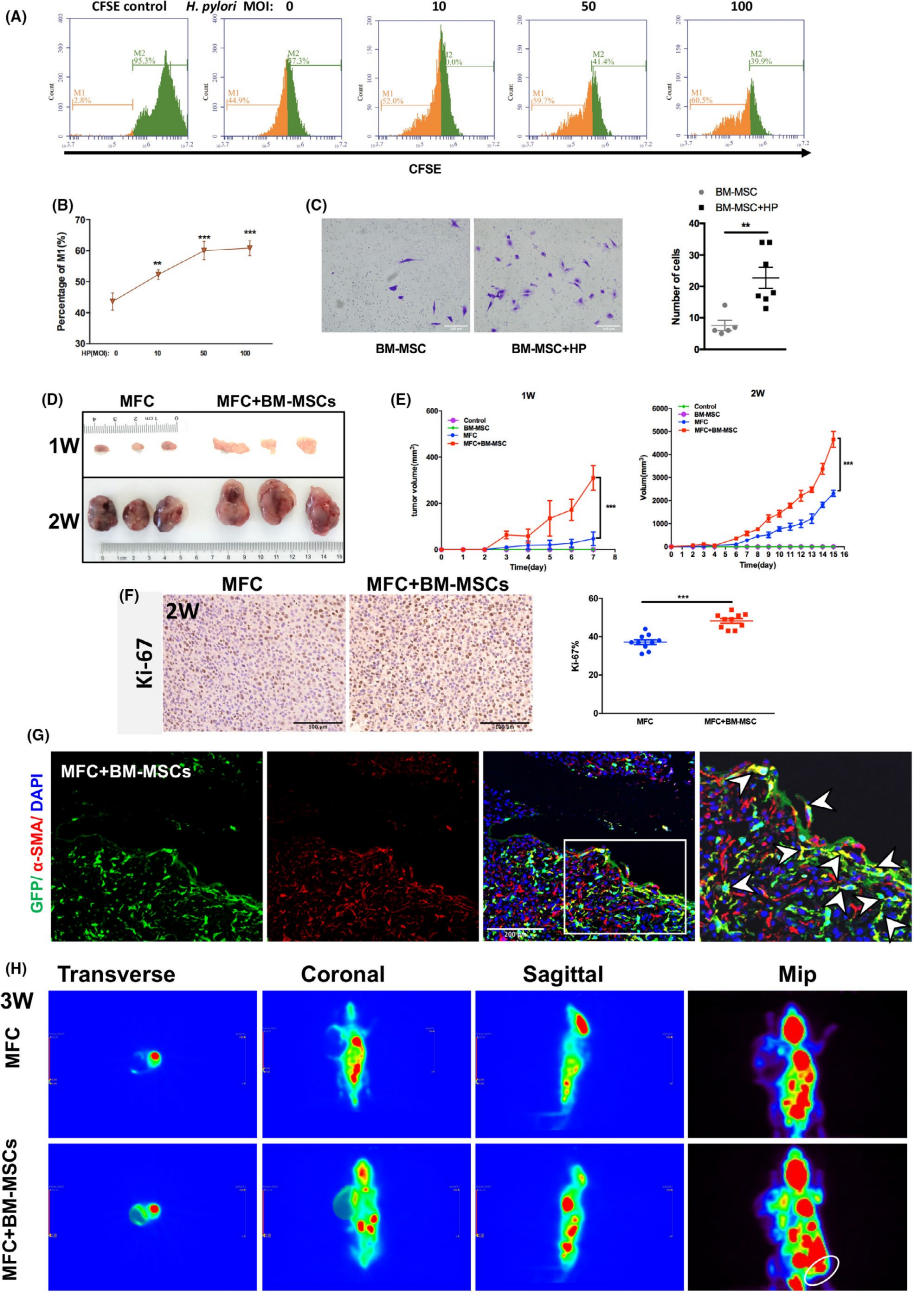
*H pylori* enhances the proliferation and migration of BM‐MSCs in vitro and promotes tumour growth in nude mice. A, Representative CFSE‐labelled BM‐MSCs after coculture with the supernatant of *H pylori* (MOI: 0, 10, 50, 100, 12 h). B, Percentage of cells in M1 in in vitro coculture with the supernatant of *H pylori* (MOI: 0, 10, 50, 100, 12 h). C, Transwell migration assay of BM‐MSCs cocultured in vitro with *H pylori* lysed by ultrasonication (MOI: 50, 18 h). D, Representative images of tumours in nude mice 1 or 2 weeks after injection of MFCs alone or co‐injection of MFCs and BM‐MSCs. E, Tumour size in the control, BM‐MSCs, MFC and MFC +BM‐MSCs groups (n = 6; *** *P* <.001). F, Representative Ki‐67 IHC images and quantitative analysis of the Ki‐67 index of tumour sections from the MFC and MFC +BM‐MSCs groups are shown. *** *P* <.001. G, Dual immunofluorescence analysis of GFP (green) and α‐SMA (red) in the MFC +BM‐MSCs group. Nuclei were labelled with DAPI (blue). H, BM‐MSCs promote the metastasis of gastric cancer xenograft tumours in nude mice. Representative PET‐CT images of nude mice 3 weeks after injection of MFCs alone or co‐injection of MFCs with BM‐MSCs in the right armpit are shown. The white circles indicate suspected abdominal metastasis of subcutaneous xenograft tumours in the MFC +BM‐MSCs group at 3 weeks post‐transplantation. Mip, Maximal Intensity Projection. Results are expressed as means ±SD. **P <.01, ***P <.001

Next, the effects of *H pylori* on the proliferation and migration of BM‐MSCs were evaluated. Proliferation was assessed using the 5‐(and‐6)‐carboxyfluorescein diacetate succinimidyl ester (CFSE) dilution assay, in which the fluorescence intensity of CFSE is halved with each successive cell division. The results of the CFSE assay showed that as the concentration of *H pylori* supernatant increased (from MOI 0 to 100), the proliferation rate of BM‐MSCs (M1, as shown in Figure 2A) increased in culture in vitro (*P* <.01; Figure 2B). The effect of *H pylori* on BM‐MSC migration was evaluated by the Transwell migration assay. The results showed that *H pylori* promoted the migration ability of BM‐MSCs in vitro (*P* <.01; Figure 2C).

### 3.4 BM‐MSCs promote the progression and metastasis of gastric cancer in nude mice xenografts

The role of BM‐MSCs in the tumorigenesis and metastasis of GC was evaluated in xenografts in nude mice. Compared with the MFC group, in which only MFC GC cells were transplanted, the subcutaneous tumour volume (Figure 2D‐E; *P* <.001) and the Ki‐67 proliferation index (Figure 2F; *P* <.001) were significantly greater in the MFC +BM‐MSCs group. Laser confocal immunofluorescence analysis of protein co‐expression in the subcutaneous tumours also showed that in the MFC +BM‐MSCs group, GFP‐labelled BM‐MSCs differentiated into α‐SMA+cells (red) (Figure 2G).

To evaluate the effect of BM‐MSCs on the metastasis of GC xenografts in nude mice, micro PET‐CT was used to detect distant metastasis. Two weeks after transplantation, no distant metastasis was observed in the control group, MFC group or MFC +BM‐MSCs group (Figure S3). Three weeks after subcutaneous transplantation, abdominal metastasis was detected in the MFC +BM‐MSCs group (white circle; Figure 2H), while no obvious distant metastasis was observed in the MFC group. Taken together, these results suggest that BM‐MSCs can promote GC progression and metastasis and can differentiate into α‐SMA+CAFs.

### 3.5 *H pylori* stimulates THBS2 protein secretion by BM‐MSCs

**FIGURE 3:**
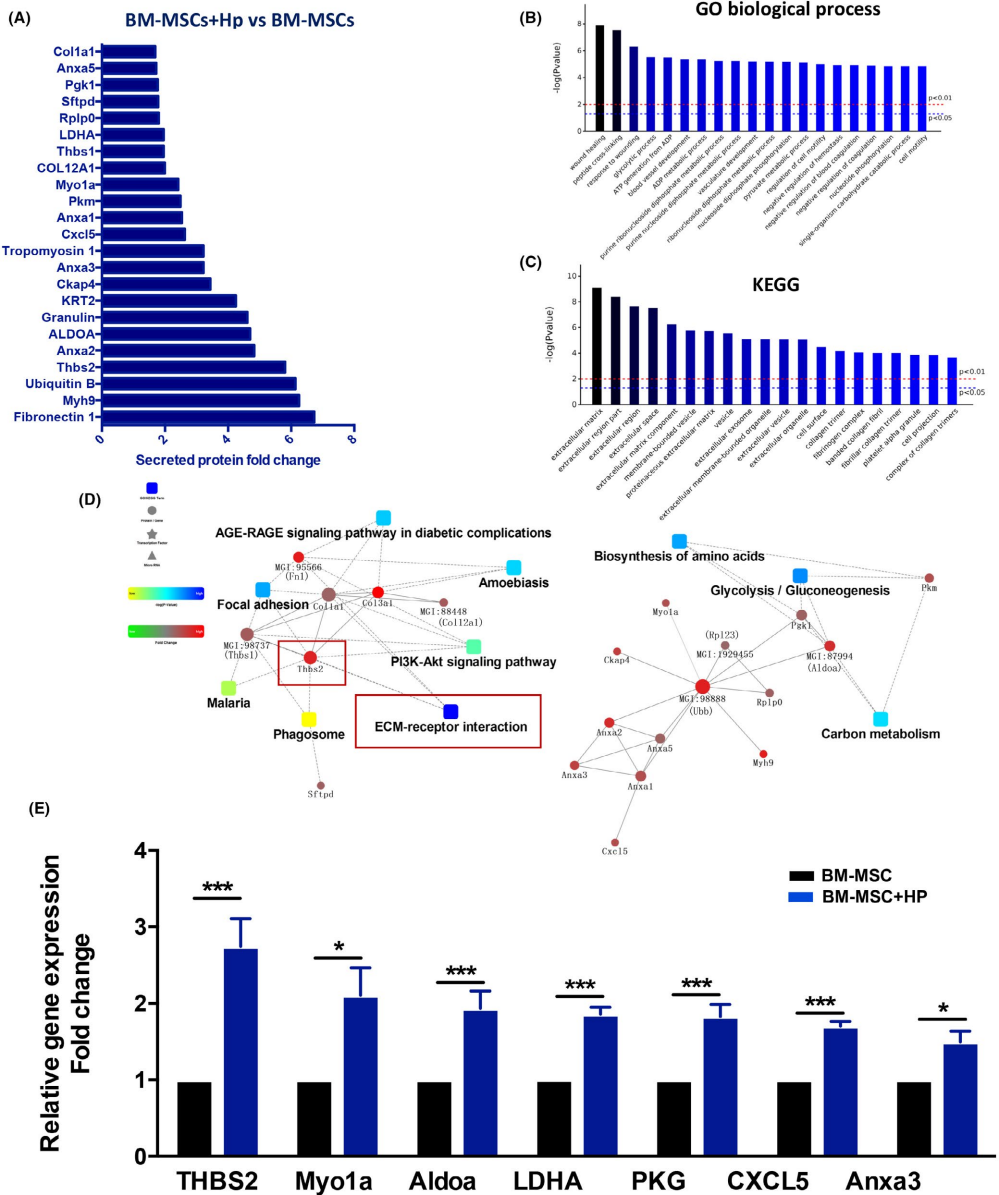
BM‐MSCs secrete the ECM‐related protein THBS2 after coculture with *H pylori*. A, Significantly upregulated proteins (fold change >1.5, *P* <.05) in BM‐MSCs upon exposure to *H pylori* were detected by iTRAQ proteomics analysis. B, The top 20 results of biological process enrichment. C, The top 20 results of regulatory pathway enrichment. D, Protein‐protein interaction analysis of the enriched KEGG and regulatory pathways. E, qRT‐PCR verification of the results of iTRAQ proteomics screening of CAF‐related genes in BM‐MSCs exposed to *H pylori*. Results are expressed as means ±SD. *P <.05, ** *P* <.01, ***P <.001

Upon *H pylori* infection, BM‐MSCs are recruited to the injured stomach and exhibit the α‐SMA+phenotype. However, the effects of the *H pylori* infection microenvironment on BM‐MSCs are not clear. Studies have confirmed that CAFs mainly promote tumour occurrence and development by secreting cytokines.^32^, ^33^, ^34^, ^35^ To identify proteins potentially associated with the occurrence of GC, iTRAQ protein quantification analysis was performed to detect the proteins secreted by BM‐MSCs upon exposure to *H pylori*. The results showed that 23 proteins were upregulated in BM‐MSCs after exposure to *H pylori* compared with the untreated group (fold change >1.5, *P* <.05; Figure 3A). According to GO biological process analysis, KEGG pathway analysis and protein‐protein interaction analysis, the secreted proteins were mainly related to the extracellular matrix (ECM) (Figure 3B‐D). qRT‐PCR analysis of the expression of CAFs‐related genes in BM‐MSCs after *H pylori* exposure showed that the mRNA level of THBS2 was most significantly upregulated (Figure 3E).

### 3.6 Depletion of the THBS2 gene reduces the tumour‐promoting ability of BM‐MSCs

**FIGURE 4:**
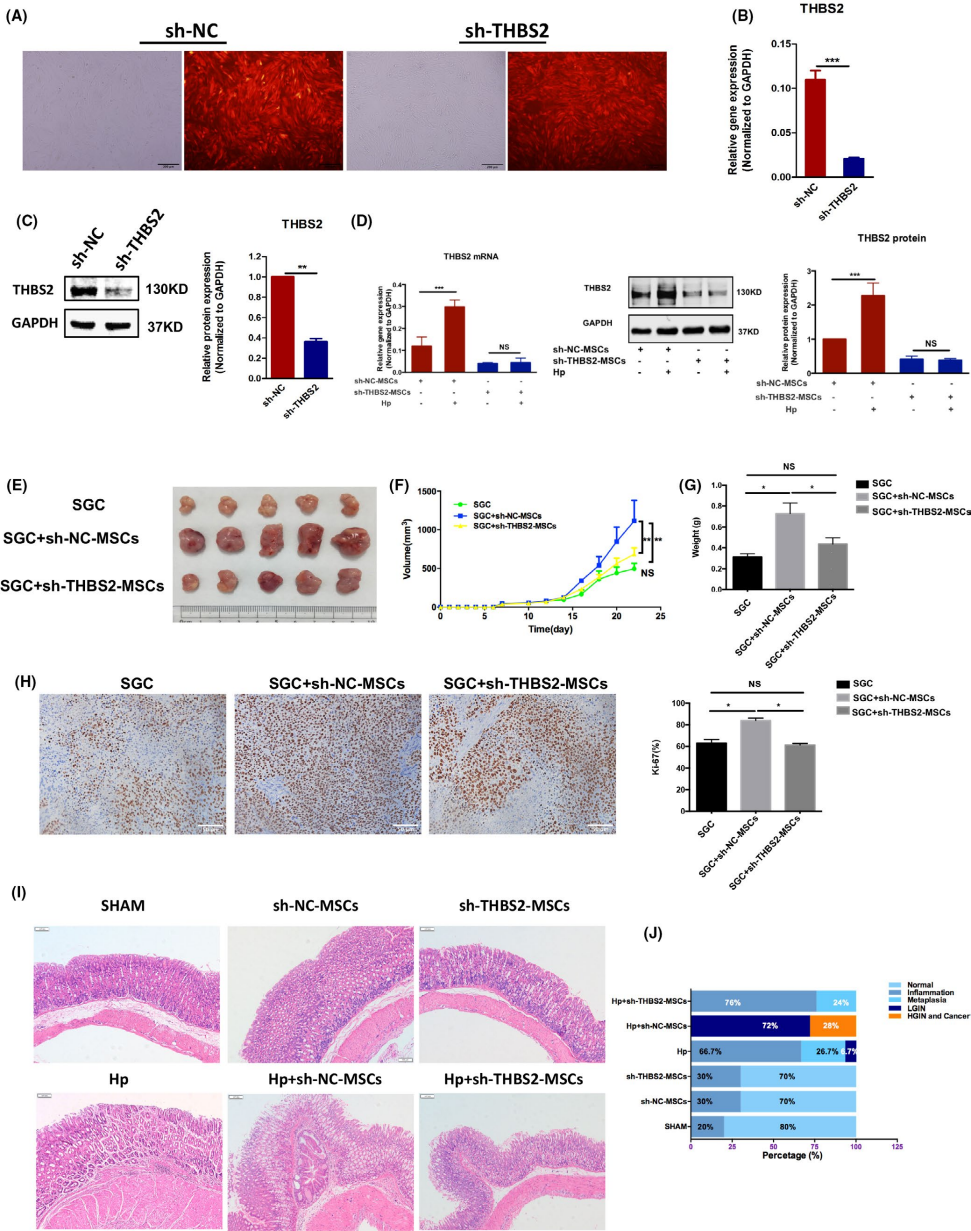
Depletion of THBS2 reduces the tumour‐promoting ability of BM‐MSCs in both gastric cancer xenografts in nude mice and chronic *H pylori*‐infected mice. A, BM‐MSCs were transfected with scrambled shRNA (negative control) or THBS2 shRNA. Transfection was confirmed by the presence of red fluorescent protein. B, RT‐qPCR confirmation of successful THBS2 gene depletion following THBS2‐specific shRNA transfection (sh‐THBS2) compared with NC transfection (sh‐NC). C, Western blotting confirmation of successful THBS2 depletion following THBS2‐specific shRNA transfection (sh‐THBS2) compared with NC transfection (sh‐NC). D, The effect of exposure to *H pylori* (MOI: 50, 12 h) on the mRNA or protein expression of BM‐MSCs transfected with scrambled shRNA or THBS2 shRNA. E, Representative tumour images from nude mice 2 weeks after injection with SGCs alone, co‐injection with SGCs and sh‐NC‐BM‐MSCs, or co‐injection with SGCs and sh‐THBS2‐BM‐MSCs. F‐G, Tumour size and tumour weight in the SGC (Control), SGC +sh‐NC‐BM‐MSCs and SGC +sh‐THBS2‐BM‐MSCs groups (n = 5 per group). H, IHC analysis of Ki‐67 expression in tumour sections from the SGC, SGC +sh‐NC‐BM‐MSCs and SGC +sh‐THBS2‐BM‐MSCs groups. I, Representative images of gastric mucosa histopathology in the SHAM, sh‐NC‐BM‐MSCs, sh‐THBS2‐BM‐MSCs, Hp, Hp +sh‐NC‐BM‐MSCs and Hp +sh‐THBS2‐BM‐MSCs groups. J, Statistics of histopathological analysis of the SHAM, sh‐NC‐BM‐MSCs, sh‐THBS2‐BM‐MSCs, Hp, Hp +sh‐NC‐BM‐MSCs and Hp +sh‐THBS2‐BM‐MSCs groups. The results are representative of at least three independent experiments. Results are expressed as means ±SD. NS, not significant, *P <.05, ** *P* <.01, *** *P* <.001

Next, we explored the effects of THBS2 expression in BM‐MSCs on GC progression. To this end, we transfected BM‐MSCs with THBS2‐specific shRNA (Figure 4A) and verified that THBS2 gene/protein expression was downregulated compared with the control group transfected with scrambled shRNA (*P* <.001; Figure 4B‐C). After exposure to *H pylori*, THBS2 mRNA and protein expression were significantly upregulated in BM‐MSCs (*P* <.001; Figure 4D) but not THBS2‐depleted BM‐MSCs (*P* >.05; Figure 4D).

In addition, our data showed that THBS2‐depleted BM‐MSCs had lower tumour‐promoting ability in GC xenografts in nude mice compared with BM‐MSCs without THBS2 knockdown (Figure 4E‐H). Similarly, in chronic *H pylori*‐infected mice, the incidence of GC was significantly lower in the Hp +sh‐THBS2‐MSCs group than in the Hp +sh‐NC‐MSCs group (0% vs. 28%, *P* <.01; Figure 4I‐J).

### 3.7 Migration of allogeneic bone marrow‐derived cells to the stomach in a patient with *H pylori* infection

**FIGURE 5:**
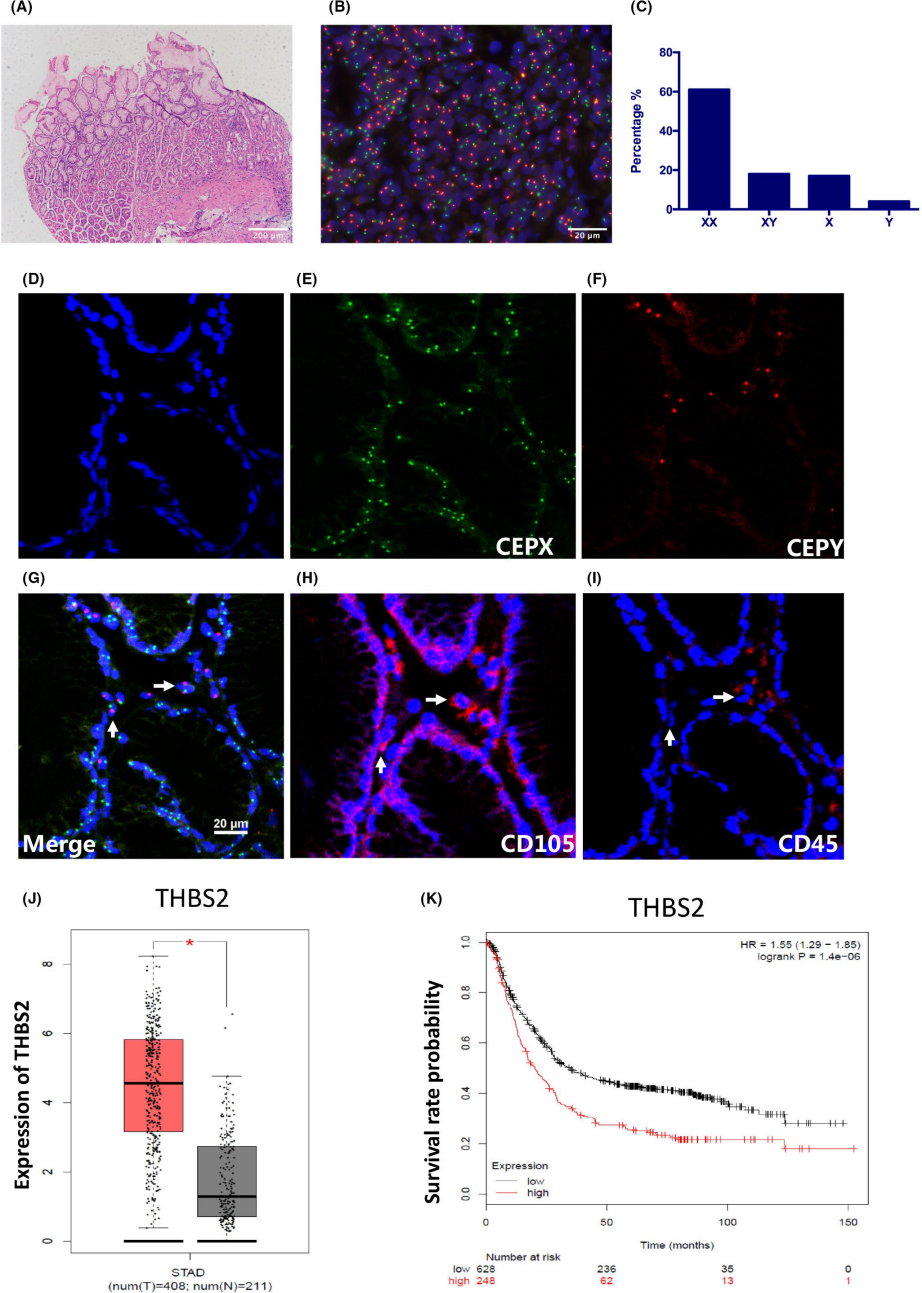
Migration of allogeneic bone marrow‐derived cells to the stomach of a patient with *H pylori‐*associated chronic gastritis. A, HE staining of the gastric biopsy of a female patient with acute myeloid leukaemia who received a bone marrow transplant from a male donor. B, Fluorescence in situ hybridization (FISH) analysis of the gastric biopsy tissue from the same patient using CEPY (orange)/CEPX (green) dual‐colour probes. C, Quantitative chromosome analysis by FISH. D‐I, FISH analysis of localized areas of the gastric gland with immunofluorescent staining of CD105 (red) or CD45 (red). Y chromosome‐positive, CD105‐positive and CD45‐negative cells (white arrows) around epithelial glands correspond to BM‐MSCs. J, Data from The Cancer Genome Atlas showed that THBS2 gene expression is significantly higher in gastric tumour tissues (T, n = 408) than in normal tissues (N, n = 211). K, Survival analysis showed that patients with gastric cancer have poorer prognosis and a lower survival rate when THBS2 expression is high [HR =1.55 (1.29‐1.85, *P* = 1.4e‐06)]. *P <.05

Analysis of the gastric mucosal tissues of a female patient with acute myeloid leukaemia who received a BM transplant from a male donor confirmed chronic gastritis (Figure 5A). Y‐chromosome‐positive cells were detected in the gastric tissue by FISH (Figure 5B). Among 100 cells examined by FISH, 61% were XX chromosome cells, 17% were X chromosome cells, 18% were XY chromosome cells, and 4% were Y chromosome cells (Figure 5C). These results suggest that BMDCs are recruited to the stomach upon *H pylori* infection. STR PCR analysis further revealed mixed chimerism in the gastric tissues from the female patient (Figure S4), with a chimerism rate of 53.4% (Table S2). To characterize the nature of the XY chromosome cells (BMDCs), a combination of FISH and laser confocal immunofluorescence microscopy was used. The result showed that the BMDCs were positive for CD105 (a marker of BM‐MSCs) (Figure 5D‐I). Taken together, these results show that allogeneic BMDCs, including BM‐MSCs, can migrate to the stomach under chronic *H pylori* infection.

### 3.8 Gastric cancer patients with high THBS2 expression have worse prognosis

Data from The Cancer Genome Atlas further confirmed that THBS2 gene expression is significantly higher in GC tissues than in normal tissues (*P* <.05, Figure 5J). Survival analysis showed that GC patients with high THBS2 expression had poorer prognosis and a lower survival rate [HR =1.55 (1.29‐1.85), *P* = 1.4e‐06; Figure 5K].

## 4 DISCUSSION

The mechanisms by which BM‐MSCs promote the occurrence and development of chronic *H pylori*‐associated GC in mice remain unclear. In this study, we established a mouse model of chronic *H pylori* infection with BM‐MSCs transplantation and demonstrated that BM‐MSCs promote the development of *H pylori*‐associated GC by differentiating into pan‐CK+epithelial cells and α‐SMA+CAFs by secreting the protein THBS2. Furthermore, we verified that BMDCs, including BM‐MSCs, can migrate to the stomach under chronic *H pylori* infection. The present study provides strong evidence that BM‐MSCs differentiate into epithelial cells and CAFs to participate in the development of *H pylori*‐associated GC in mice.

Previous studies have shown that BM‐MSCs are recruited to various types of tumour tissues.^23^ We found that transplanted BM‐MSCs gradually migrated to the mucosa and promoted the development of GC in chronic *H pylori*‐infected mice but not in mice without *H pylori* infection. We hypothesize that the microenvironment of chronic *H pylori* infection might recruit BM‐MSCs to the injured mucosa, thereby promoting the development of GC. We also confirmed the migration of BMDCs in a human patient. This patient, a woman with acute myeloid leukaemia, chronic gastritis and *H pylori* infection, received a bone marrow transplant from a male donor. We found a high chimerism rate in the gastric mucosal tissue from the patient one year after transplantation, as well as XY chromosome cells. The combination of FISH and laser confocal immunofluorescence microscopy was further used to analyse the nature of the XY chromosome cells (BMDCs) in gastric tissue, and the result showed that the BMDCs were CD105 positive and CD45 negative. As known, CD105 is a marker of BM‐MSCs, and the CD45 is a marker of BM‐derived leukocytes. But there is also the possibility that the migratory cells are leukaemia cells internalizing the donor's male Y chromosome through the process of cell fusion. On the whole, our results suggest that BMDCs, most likely BM‐MSCs, can migrate to the stomach in the microenvironment of chronic *H pylori* infection, which may explain the involvement of BMDCs in the occurrence and development of chronic *H pylori*‐associated GC.

Although some studies have confirmed that BM‐MSCs are involved in the occurrence of GC, there is no consensus on whether BM‐MSCs differentiate into gastric epithelial cells or CAFs.^17^, ^18^ Consequently, we examined the specific role and mechanism of BM‐MSCs in *H pylori‐*associated GC. We found that BM‐MSCs differentiated into both epithelial cells and CAFs in mice with chronic *H pylori* infection, thus both supporting and explaining the conflicting results found in the previous literature.^17^, ^18^

We also explored the mechanism of BM‐MSCs as CAFs in *H pylori‐*associated GC. THBS2 has been linked to a variety of diseases.^39^, ^40^, ^41^, ^42^, ^43^, ^44^, ^45^ Cao et al and Li et al both found that THBS2 gene expression is significantly upregulated in GC and that patients with high THBS2 expression have poorer prognosis.^46^, ^47^ Zhou et al found that THBS2 gene expression is positively correlated with colon cancer development and TNM stage.^48^ In our study, the results of proteomic iTRAQ analysis and further verification confirmed that as CAFs, BM‐MSCs secrete the protein THBS2 to promote the development of GC induced by chronic *H pylori* infection.

In summary, several key findings of this study should be emphasized. First, we confirmed that BMDCs, including BM‐MSCs, migrate to the *H pylori‐*infected stomach. Second, BM‐MSCs differentiate into epithelial cells and CAFs in chronic *H pylori‐*infected mice. Third, BM‐MSCs secrete the protein THBS2 to promote the progression of chronic *H pylori*‐associated GC. Therefore, THBS2 may be a potential therapeutic target for chronic *H pylori‐*associated GC.

## CONFLICTS OF INTEREST

None.

## AUTHORS CONTRIBUTIONS

Rong Lin designed and supervised the study and data analysis; Huiying Shi, Cuihua Qi and Lingjun Meng performed most of experiments, analysed the data, wrote and revised the manuscript; Hailing Yao, Chen Jiang and Mengke Fan helped the experiments and analysed the data; Qin Zhang provided pathological assessment and analysis; Xiaohua Hou supervised the study. All the authors approved the final manuscript and agreed for the publication.

## ETHICAL APPROVAL

All procedures followed were in accordance with the ethical standards of the responsible committee on human experimentation (institutional and national) and with the Helsinki Declaration of 1964 and later versions. Informed consent was obtained from the patient included in the study. All institutional and national guidelines for the care and use of laboratory animals were followed.

## Supporting information

Figure S1

Figure S2

Figure S3

Figure S4

Table S1‐S2

Supporting information

## Data Availability

Main data generated or analysed during this study are included in this published article, and detailed data are available from the corresponding author on reasonable request.

## References

[1] Sung H, Ferlay J, Siegel RL, et al. Global Cancer Statistics 2020: GLOBOCAN Estimates of Incidence and Mortality Worldwide for 36 Cancers in 185 Countries. Cancer J Clin. 2021;71(3):209‐249. 10.3322/caac.21660 33538338

[2] Bae JM. Sex as an effect modifier in the association between alcohol intake and gastric cancer risk. World J Gastrointest Oncol. 2021;13(5):453‐461. 34040705 10.4251/wjgo.v13.i5.453 PMC8131903

[3] Deng W, Jin L, Zhuo H, Vasiliou V, Zhang Y. Alcohol consumption and risk of stomach cancer: A meta‐analysis. Chem Biol Interact. 2021;336:109365. 33412155 10.1016/j.cbi.2021.109365

[4] Butt J, Varga MG, Wang T, et al. Smoking, Helicobacter Pylori Serology, and Gastric Cancer Risk in Prospective Studies from China, Japan, and Korea. Cancer Prev Res (Phila). 2019;12(10):667‐674. 31350279 10.1158/1940-6207.CAPR-19-0238 PMC6854526

[5] Lyons K, Le LC, Pham YT, et al. Gastric cancer: epidemiology, biology, and prevention: a mini review. Eur J Cancer Prev. 2019;28(5):397‐412. 31386635 10.1097/CEJ.0000000000000480

[6] Strumylaite L, Zickute J, Dudzevicius J, Dregval L. Salt‐preserved foods and risk of gastric cancer. Medicina (Kaunas). 2006;42(2):164‐170. 16528133

[7] Lin SH, Li YH, Leung K, Huang CY, Wang XR. Salt processed food and gastric cancer in a Chinese population. Asian Pac J Cancer Prev. 2014;15(13):5293‐5298. 25040991 10.7314/apjcp.2014.15.13.5293

[8] Bray F, Ferlay J, Soerjomataram I, Siegel RL, Torre LA, Jemal A. Global cancer statistics 2018: GLOBOCAN estimates of incidence and mortality worldwide for 36 cancers in 185 countries. Cancer J Clin. 2018;68(6):394–424. 10.3322/caac.21492 30207593

[9] Plummer M, Franceschi S, Vignat J, Forman D, de Martel C. Global burden of gastric cancer attributable to Helicobacter pylori. Int J Cancer. 2015;136(2):487‐490. 24889903 10.1002/ijc.28999

[10] Javed S, Skoog EC, Solnick JV. Impact of Helicobacter pylori Virulence Factors on the Host Immune Response and Gastric Pathology. Curr Top Microbiol Immunol. 2019;421:21‐52. 31123884 10.1007/978-3-030-15138-6_2

[11] Baj J, Korona‐Glowniak I, Forma A, et al. Mechanisms of the Epithelial‐Mesenchymal Transition and Tumor Microenvironment in Helicobacter pylori‐Induced Gastric Cancer. Cells. 2020;9(4):1055. 10.3390/cells9041055 PMC7225971 32340207

[12] Correa P, Haenszel W, Cuello C, Tannenbaum S, Archer M. A model for gastric cancer epidemiology. Lancet. 1975;2(7924):58‐60. 49653 10.1016/s0140-6736(75)90498-5

[13] Chiba T, Marusawa H, Ushijima T. Inflammation‐associated cancer development in digestive organs: mechanisms and roles for genetic and epigenetic modulation. Gastroenterology. 2012;143(3):550‐563. 22796521 10.1053/j.gastro.2012.07.009

[14] Correa P, Houghton J. Carcinogenesis of Helicobacter pylori. Gastroenterology. 2007;133(2):659‐672. 17681184 10.1053/j.gastro.2007.06.026

[15] Peek RM Jr, Moss SF, Tham KT, et al. Helicobacter pylori cagA+ strains and dissociation of gastric epithelial cell proliferation from apoptosis. J Natl Cancer Inst. 1997;89(12):863‐868. 9196252 10.1093/jnci/89.12.863

[16] Xia HH, Talley NJ. Apoptosis in gastric epithelium induced by Helicobacter pylori infection: implications in gastric carcinogenesis. Am J Gastroenterol. 2001;96(1):16‐26. 11197247 10.1111/j.1572-0241.2001.03447.x

[17] Houghton J, Stoicov C, Nomura S, et al. Gastric cancer originating from bone marrow‐derived cells. Science. 2004;306(5701):1568‐1571. 15567866 10.1126/science.1099513

[18] Quante M, Tu SP, Tomita H, et al. Bone marrow‐derived myofibroblasts contribute to the mesenchymal stem cell niche and promote tumor growth. Cancer Cell. 2011;19(2):257‐272. 21316604 10.1016/j.ccr.2011.01.020 PMC3060401

[19] Zhao FY, Cheng TY, Yang L, et al. G‐CSF Inhibits Pulmonary Fibrosis by Promoting BMSC Homing to the Lungs via SDF‐1/CXCR4 Chemotaxis. Sci Rep. 2020;10(1):10515. 32601321 10.1038/s41598-020-65580-2 PMC7324625

[20] Wang J, Zhu L, Chen X, Huang R, Wang S, Dong P. Human Bone Marrow Mesenchymal Stem Cells Functionalized by Hybrid Baculovirus‐Adeno‐Associated Viral Vectors for Targeting Hypopharyngeal Carcinoma. Stem Cells Dev. 2019;28(8):543‐553. 30747033 10.1089/scd.2018.0252

[21] Meng L, Zhao Y, Bu W, et al. Bone mesenchymal stem cells are recruited via CXCL8‐CXCR2 and promote EMT through TGF‐beta signal pathways in oral squamous carcinoma. Cell Prolif. 2020;53(8):e12859. 32588946 10.1111/cpr.12859 PMC7445409

[22] Zheng XB, He XW, Zhang LJ, et al. Bone marrow‐derived CXCR4‐overexpressing MSCs display increased homing to intestine and ameliorate colitis‐associated tumorigenesis in mice. Gastroenterol Rep (Oxf). 2019;7(2):127‐138. 30976426 10.1093/gastro/goy017 PMC6454852

[23] Nishikawa G, Kawada K, Nakagawa J, et al. Bone marrow‐derived mesenchymal stem cells promote colorectal cancer progression via CCR5. Cell Death Dis. 2019;10(4):264. 30890699 10.1038/s41419-019-1508-2 PMC6424976

[24] Takigawa H, Kitadai Y, Shinagawa K, et al. Mesenchymal stem cells induce epithelial to mesenchymal transition in colon cancer cells through direct cell‐to‐cell contact. Neoplasia. 2017;19(5):429‐438. 28433772 10.1016/j.neo.2017.02.010 PMC5402629

[25] Karnoub AE, Dash AB, Vo AP, et al. Mesenchymal stem cells within tumour stroma promote breast cancer metastasis. Nature. 2007;449(7162):557‐563. 17914389 10.1038/nature06188

[26] Chan TS, Shaked Y, Tsai KK. Targeting the Interplay Between Cancer Fibroblasts, Mesenchymal Stem Cells, and Cancer Stem Cells in Desmoplastic Cancers. Front Oncol. 2019;9:688. 31417869 10.3389/fonc.2019.00688 PMC6684765

[27] Ohlund D, Elyada E, Tuveson D. Fibroblast heterogeneity in the cancer wound. J Exp Med. 2014;211(8):1503‐1523. 25071162 10.1084/jem.20140692 PMC4113948

[28] Vicent S, Sayles LC, Vaka D, et al. Cross‐species functional analysis of cancer‐associated fibroblasts identifies a critical role for CLCF1 and IL‐6 in non‐small cell lung cancer in vivo. Can Res. 2012;72(22):5744‐5756. 10.1158/0008-5472.CAN-12-1097 PMC3856949 22962265

[29] Paunescu V, Bojin FM, Tatu CA, et al. Tumour‐associated fibroblasts and mesenchymal stem cells: more similarities than differences. J Cell Mol Med. 2011;15(3):635‐646. 20184663 10.1111/j.1582-4934.2010.01044.x PMC3922385

[30] Desmouliere A, Guyot C, Gabbiani G. The stroma reaction myofibroblast: a key player in the control of tumor cell behavior. Int J Dev Biol. 2004;48(5–6):509‐517. 15349825 10.1387/ijdb.041802ad

[31] Santi A, Kugeratski FG, Zanivan S. Cancer Associated Fibroblasts: The Architects of Stroma Remodeling. Proteomics. 2018;18(5–6):e1700167. 29280568 10.1002/pmic.201700167 PMC5900985

[32] Erdogan B, Webb DJ. Cancer‐associated fibroblasts modulate growth factor signaling and extracellular matrix remodeling to regulate tumor metastasis. Biochem Soc Trans. 2017;45(1):229‐236. 28202677 10.1042/BST20160387 PMC5371349

[33] Tarnowski M, Grymula K, Liu R, et al. Macrophage migration inhibitory factor is secreted by rhabdomyosarcoma cells, modulates tumor metastasis by binding to CXCR4 and CXCR7 receptors and inhibits recruitment of cancer‐associated fibroblasts. Mol Cancer Res. 2010;8(10):1328‐1343. 20861157 10.1158/1541-7786.MCR-10-0288 PMC2974061

[34] Cadamuro M, Brivio S, Mertens J, et al. Platelet‐derived growth factor‐D enables liver myofibroblasts to promote tumor lymphangiogenesis in cholangiocarcinoma. J Hepatol. 2019;70(4):700‐709. 30553841 10.1016/j.jhep.2018.12.004 PMC10878126

[35] Mishra P, Banerjee D, Ben‐Baruch A. Chemokines at the crossroads of tumor‐fibroblast interactions that promote malignancy. J Leukoc Biol. 2011;89(1):31‐39. 20628066 10.1189/jlb.0310182

[36] Beckermann BM, Kallifatidis G, Groth A, et al. VEGF expression by mesenchymal stem cells contributes to angiogenesis in pancreatic carcinoma. Br J Cancer. 2008;99(4):622‐631. 18665180 10.1038/sj.bjc.6604508 PMC2527820

[37] Spaeth EL, Dembinski JL, Sasser AK, et al. Mesenchymal stem cell transition to tumor‐associated fibroblasts contributes to fibrovascular network expansion and tumor progression. PLoS One. 2009;4(4):e4992. 19352430 10.1371/journal.pone.0004992 PMC2661372

[38] Lin R, Ma H, Ding Z, et al. Bone marrow‐derived mesenchymal stem cells favor the immunosuppressive T cells skewing in a Helicobacter pylori model of gastric cancer. Stem Cells Dev. 2013;22(21):2836‐2848. 23777268 10.1089/scd.2013.0166

[39] Wang HQ, Jian T, Wang F, Wang X. Impact of thrombospondin‐2 gene variations on the risk of thoracic aortic dissection in a Chinese Han population. Int J Clin Exp Med. 2014;7(12):5796‐5801. 25664109 PMC4307556

[40] Hyun SJ, Park BG, Rhim SC, Jang JW, Jeon SR, Roh SW. Progression of lumbar spinal stenosis is influenced by polymorphism of thrombospondin 2 gene in the Korean population. Eur Spine J. 2014;23(1):57‐63. 23807322 10.1007/s00586-013-2866-6 PMC3897815

[41] Berisha B, Schams D, Rodler D, Sinowatz F, Pfaffl MW. Expression and localization of members of the thrombospondin family during final follicle maturation and corpus luteum formation and function in the bovine ovary. J Reprod Dev. 2016;62(5):501‐510. 27396384 10.1262/jrd.2016-056 PMC5081738

[42] Wang Y, Fu W, Xie F, et al. Common polymorphisms in ITGA2, PON1 and THBS2 are associated with coronary atherosclerosis in a candidate gene association study of the Chinese Han population. J Hum Genet. 2010;55(8):490‐494. 20485444 10.1038/jhg.2010.53

[43] Czekierdowski A, Czekierdowska S, Danilos J, et al. Microvessel density and CpG island methylation of the THBS2 gene in malignant ovarian tumors. J Physiol Pharmacol. 2008;59(Suppl 4):53‐65. 18955754

[44] Whitcomb BP, Mutch DG, Herzog TJ, Rader JS, Gibb RK, Goodfellow PJ. Frequent HOXA11 and THBS2 promoter methylation, and a methylator phenotype in endometrial adenocarcinoma. Clinical cancer research : an official journal of the American Association for Cancer Research. 2003;9(6):2277‐2287. 12796396

[45] Hsu CW, Yu JS, Peng PH, et al. Secretome profiling of primary cells reveals that THBS2 is a salivary biomarker of oral cavity squamous cell carcinoma. J Proteome Res. 2014;13(11):4796‐4807. 24708169 10.1021/pr500038k

[46] Cao L, Chen Y, Zhang M, et al. Identification of hub genes and potential molecular mechanisms in gastric cancer by integrated bioinformatics analysis. PeerJ. 2018;6:e5180. 30002985 10.7717/peerj.5180 PMC6033081

[47] Li T, Gao X, Han L, Yu J, Li H. Identification of hub genes with prognostic values in gastric cancer by bioinformatics analysis. World J Surg Oncol. 2018;16(1):114. 29921304 10.1186/s12957-018-1409-3 PMC6009060

[48] Zhou XG, Huang XL, Liang SY, et al. Identifying miRNA and gene modules of colon cancer associated with pathological stage by weighted gene co‐expression network analysis. OncoTargets and therapy. 2018;11:2815‐2830. 29844680 10.2147/OTT.S163891 PMC5961473

