# Supplementary figures and images for: Bone marrow‐derived mesenchymal stem cells promote Helicobacter pylori‐associated gastric cancer progression by secreting thrombospondin‐2
Source: Cell Prolif. 2021 Aug 25;54(10):e13114. doi: 10.1111/cpr.13114 (PMC8488559; doi:10.1111/cpr.13114)

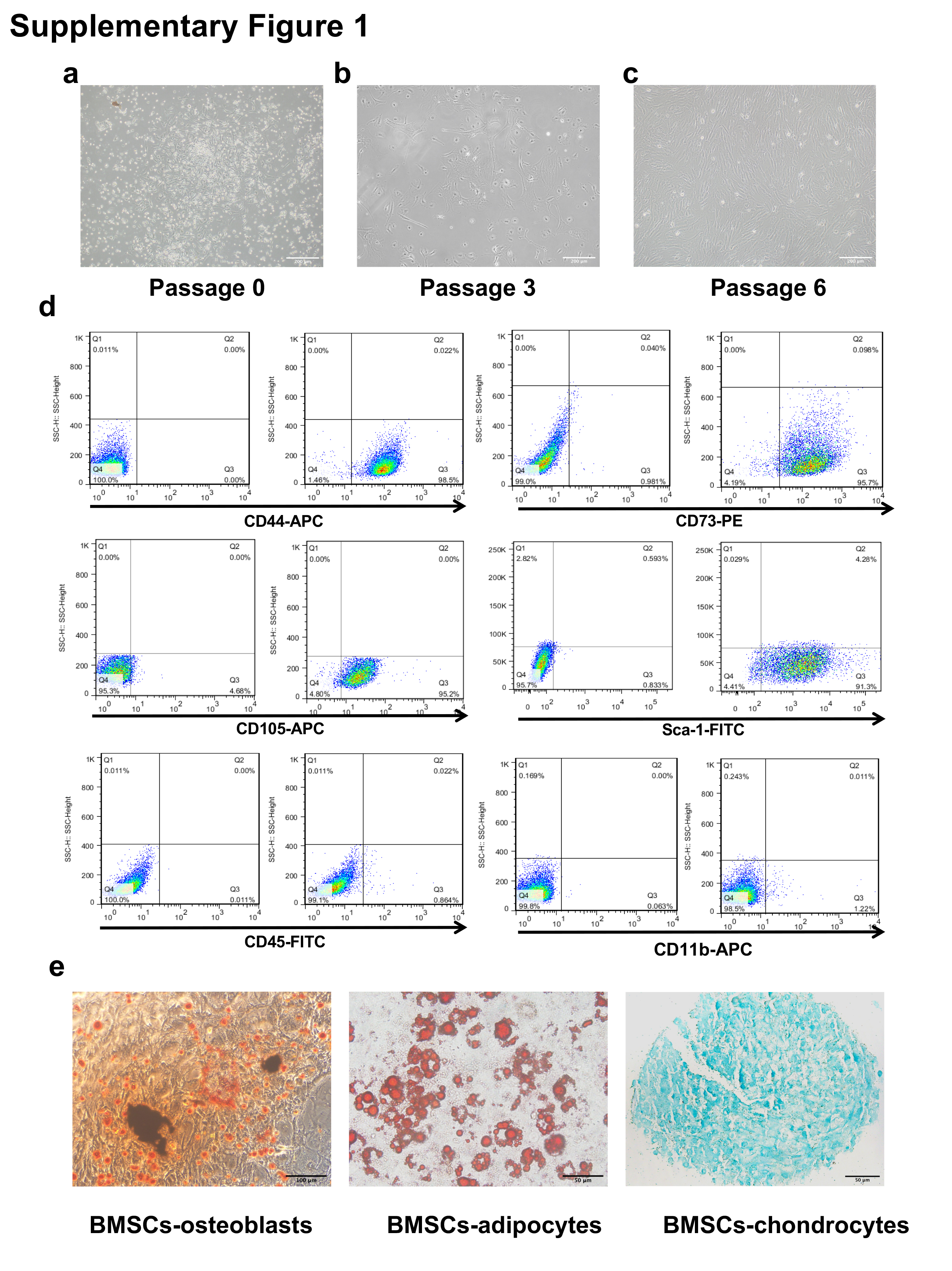

Supplement: Supplementary file 1 — Figure S1 [file CPR-54-e13114-s003.jpg]

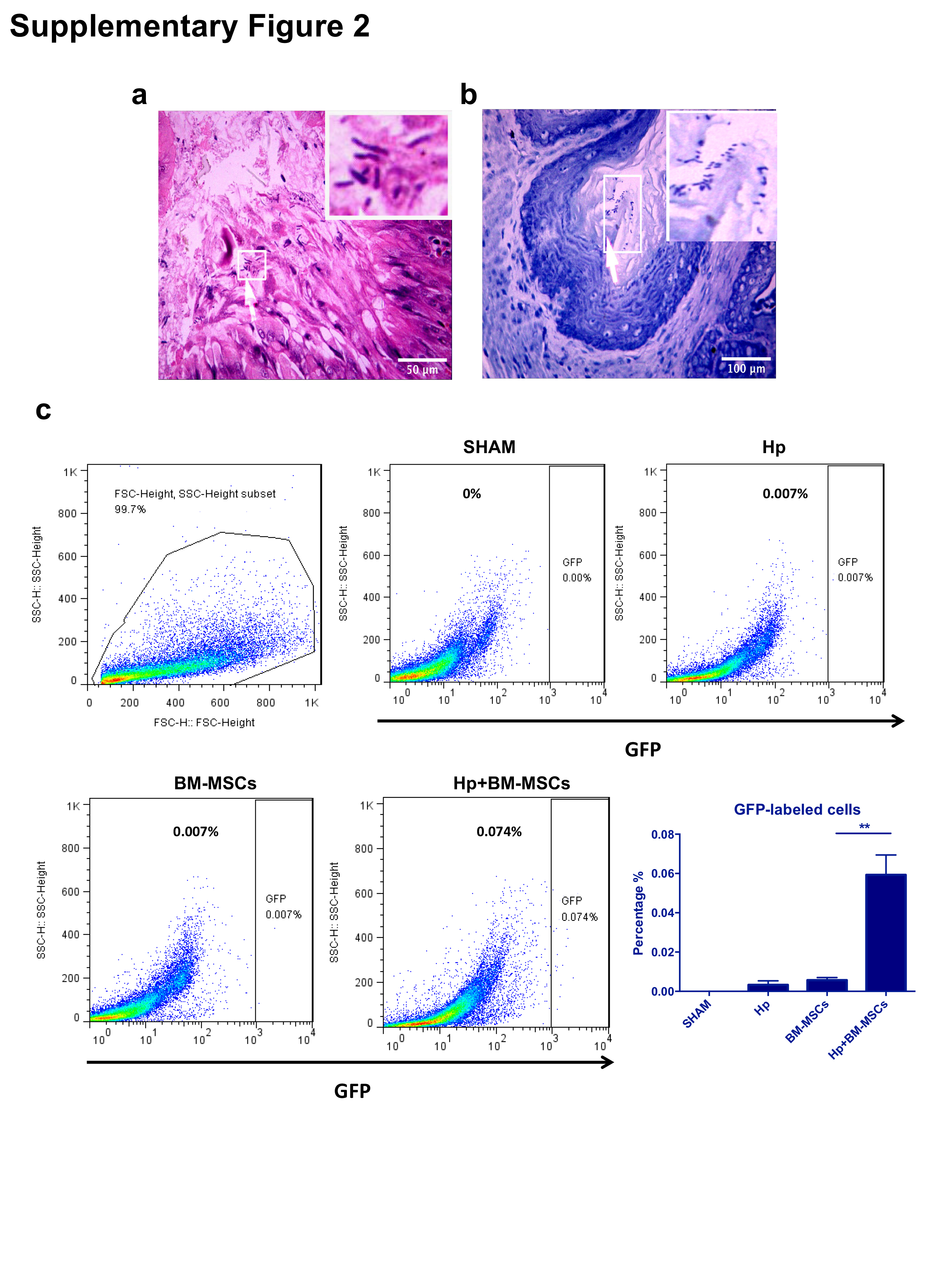

Supplement: Supplementary file 2 — Figure S2 [file CPR-54-e13114-s001.jpg]

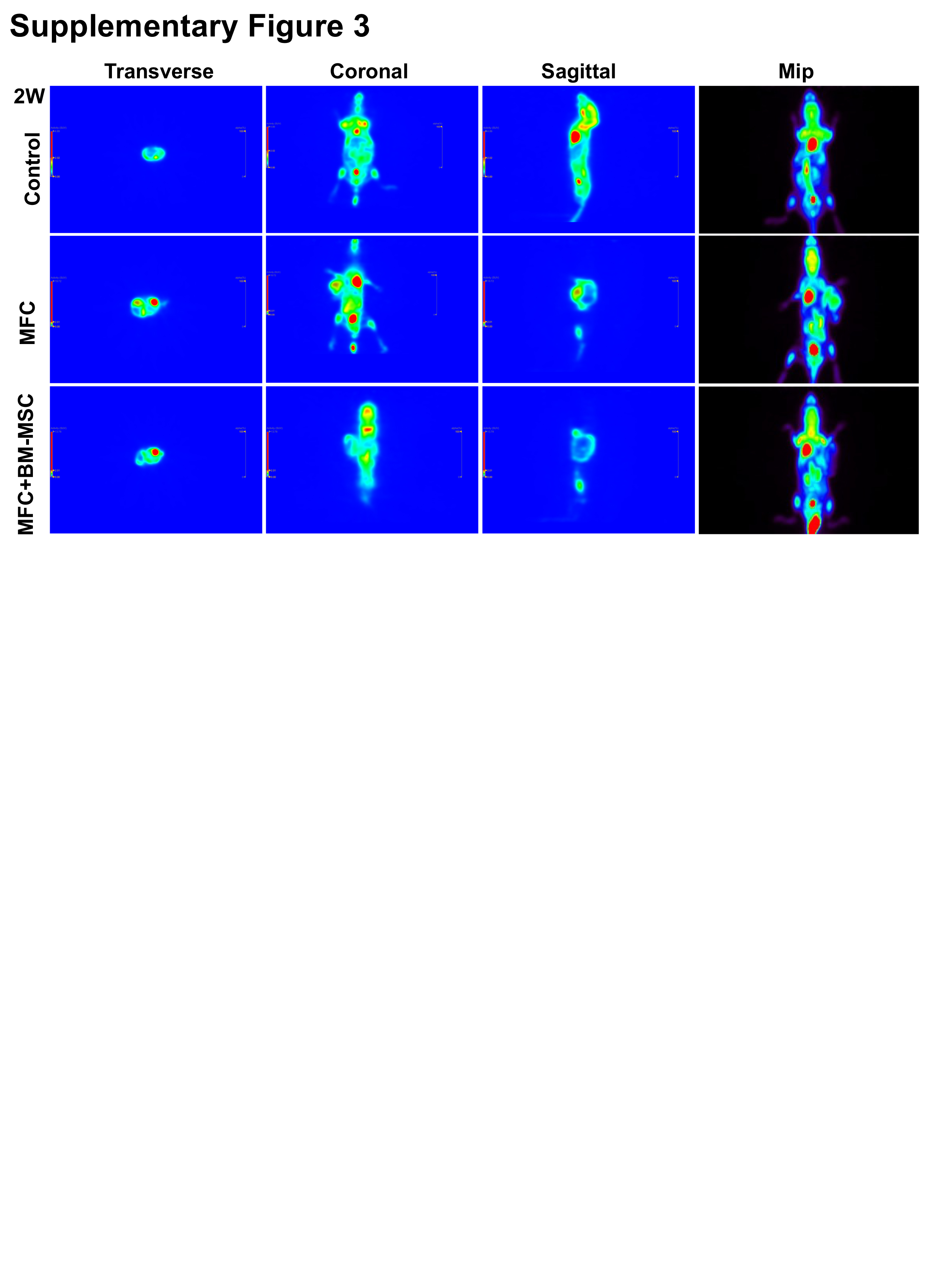

Supplement: Supplementary file 3 — Figure S3 [file CPR-54-e13114-s005.jpg]

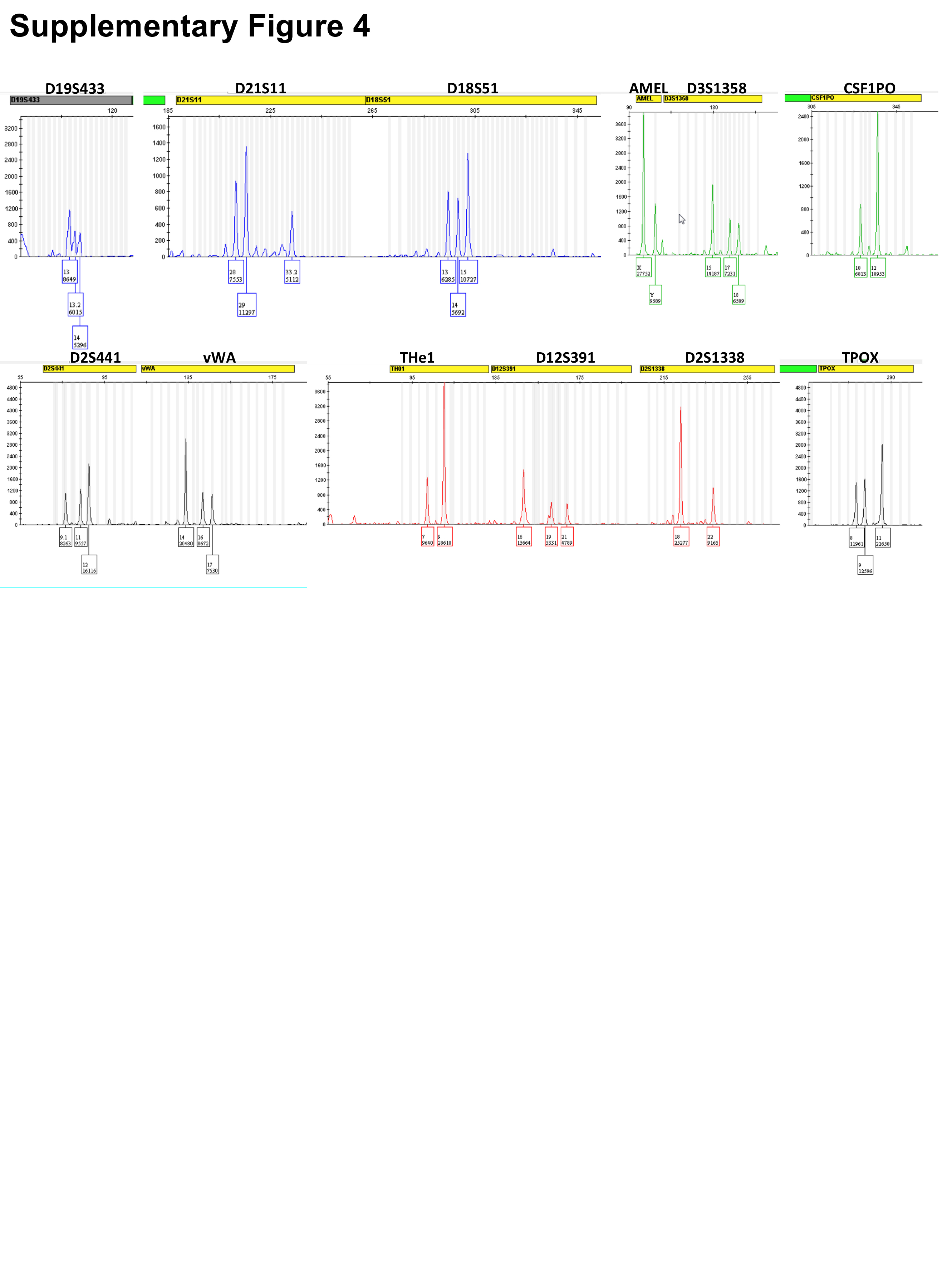

Supplement: Supplementary file 4 — Figure S4 [file CPR-54-e13114-s006.jpg]
