# Supplementary material for: Bone marrow‐derived mesenchymal stem cells promote Helicobacter pylori‐associated gastric cancer progression by secreting thrombospondin‐2
Source: Cell Prolif. 2021 Aug 25;54(10):e13114. doi: 10.1111/cpr.13114 (PMC8488559; doi:10.1111/cpr.13114)
Supplement: Supplementary file 5 — Table S1‐S2 [file CPR-54-e13114-s002.doc]

**Supplementary table 1.** Primers used for qRT-PCR assay.

| Gene | *Forward primer* | *Reverse primer* |
| --- | --- | --- |
| Fibronectin 1 | ATGGCGACGGTATTCTGTAAAG | TTGGCAGTTGGTCAATCACAT |
| Annexin A1 | CCCAGGACCACCTTTGTATG | TGACTGACCCGTAGGCACTT |
| Annexin A2 | ATGTCTACTGTCCACGAAATCCT | TGACTGACCCGTAGGCACTT |
| Annexin A3 | CGGGCTAAAGCCTGGATAAT | TGTGATCTCGGCTTGAGAGA |
| COL12A1 | CTGAGGTCTGGGTAAAGGCA | CTGGCCTCCTTCCTAACACA |
| SFTPD | CCTCTCGCAGAGATCAGTACC | CCTCTCGCAGAGATCAGTACC |
| CXCL-5 | GTTCCATCTCGCCATTCATGC | GCGGCTATGACTGAGGAAGG |
| PKG | ATGTCGCTTTCCAACAAGCTG | GCTCCATTGTCCAAGCAGAAT |
| COL1A1 | GCTCCTCTTAGGGGCCACT | TTGGGGACCCTTAGGCCAT |
| Annexin A5 | AGACGAGAAAGTATTGACCGAGA | ACCACCAACATCCTCTGGTAG |
| THBS2 | CTGGGCATAGGGCCAAGAG | GTCTTCCGGTTAATGTTGCTGAT |
| Myo1a | ATGTGGTGATCTCAATGAACCC | AGTATAGTCCCGGTATTTGGCA |
| CKAP4 | TCCCGTCAGAGGGATGAGC | GCTGGGAGTTTCTCAGGAGG |
| THBS1 | CCTGCCAGGGAAGCAACAA | ACAGTCTATGTAGAGTTGAGCCC |
| MYH9 | AGAAGTTGGTATGGGTGCCTT | CCCTGAGTAGTATCGCTCCTTG |
| ALDOA | AGTCCACCGGAAGCATTGC | CAGCCCCTGGGTAGTTGTC |
| LDHA | CAAAGACTACTGTGTAACTGCGA | TGGACTGTACTTGACAATGTTGG |
| GAPDH | AGGTCGGTGTGAACGGATTTG-3′ | GGGGTCGTTGATGGCAACA |

**Supplementary table 2. STR analysis results of the gastric biopsy of the female patient with acute myeloid leukemia receiving a bone marrow transplant from a male donor using the short tandem repeat sequence analysis.**

| **STR Marker** | **Result** |
| --- | --- |
| **D19S433** | 13/13.2/14 |
| **D5S818** | 11 |
| **D21S11** | 28/29/33.2 |
| **D18S51** | 13/14/15 |
| **D6S1043** | 11/12 |
| **AMEL** | X/Y |
| **D3S1358** | 15/17/18 |
| **D13S317** | 12/13 |
| **D7S820** | 11/12 |
| **D16S539** | 11/12 |
| **CSF1PO** | 10/12 |
| **Penta D** | 10/11 |
| **D2S441** | 9.1/11/12 |
| **vWA** | 14/16/17 |
| **D8S1179** | 11/15 |
| **TPOX** | 8/9/11 |
| **Penta E** | 11 |
| **TH01** | 7/9 |
| **D12S391** | 16/19/21 |
| **D2S1338** | 18/22 |
| **FGA** | 21/22 |
