# Supplementary material for: Bone marrow‐derived mesenchymal stem cells promote Helicobacter pylori‐associated gastric cancer progression by secreting thrombospondin‐2
Source: Cell Prolif. 2021 Aug 25;54(10):e13114. doi: 10.1111/cpr.13114 (PMC8488559; doi:10.1111/cpr.13114)
Supplement: Supplementary file 6 — Supporting information [file CPR-54-e13114-s004.docx]

**Supplementary materials and methods**

**Cell culture**

BM-MSCs were isolated from femurs of 4-6 weeks old male BALB/c mice (Beijing Huafukang Biotechnology Co., Ltd, Beijing, China). Cells were cultured in low glucose Dulbecco’s modified Eagle’s medium (DMEM, Gibco), supplemented with 10% fetal bovine serum (FBS, Gibco), 100U/ml penicillin and 100ug/ml streptomycin. The cells were placed in a humidified incubator at 37°C and 5% CO2. Nonadherent cells were removed after 24 h and the culture media was refreshed every 2-3 days. After 7-10 days, about 80% confluent cells were dissociated with 0.25% trypsin (Gibco) and passed into new culture flasks. The human gastric adenocarcinoma cell lines SGC-7901 and mouse forestomach carcinoma cell line (MFC) were purchased from Shanghai Zhongqiao Xinzhou Biological Technology Co., Ltd. (Shanghai, China) and cultured in RPMI1640 with 10% FBS at 37°C in a humidified cell incubator with 5% CO_2_.

**Characterization of BM-MSCs**

To identify the characterization of the isolated cells, cell surface markers and differential potential were examined. BM-MSCs at passage 6 were identified by staining with antibodies to CD44, CD73, CD105, CD11b, CD45, and stem cell antigen (Sca)-1. The expression of cell surface antigen was analyzed by flow cytometry. BM-MSCs at passage 6 were harvested and the cell suspension were stained with the APC-conjugated rat anti-mouse CD44, PE-conjugated rat anti-mouse CD73, APC-conjugated rat anti-mouse CD105, FITC-conjugated rat anti-mouse Sca-1, FITC-conjugated rat anti-mouse CD45, and APC-conjugated rat anti-mouse CD11b (BD Pharmingen, Franklin Lakes, NJ, USA) for 1 hour at 4°C. After washing with PBS, staining buffer was added and the cells were used for flow cytometric analysis.

For osteogenic differentiation, BM-MSCs at passage 6 were dissociated with 0.25% trypsin and the cell suspension containing 2.0×10^5^ cells were seeded into the gelatin-coated 6-well plates. Then cells were cultured using OriCellTM Balb/c Mouse Bone Marrow Mesenchymal Stem Cell Osteogenic Differentiation Medium Kit (Cyagen, Silicon Valley, CA, USA). After 3 weeks, the medium was removed and cells were fixed in 4% paraformaldehyde for 10 min and stained with Alizarin red dye (Cyagen, Silicon Valley, CA, USA). For adipogenic differentiation, BM-MSCs at passage 6 were seeded into the gelatin-coated 6-well plates and cultured in Adipogenic Differentiation Medium Kit (Cyagen, Silicon Valley, CA, USA) for 4 weeks according to the manufacturer's instructions. Oil red-O solution was used for the assessment of the presence of lipid-rich vacuoles. For chondrogenic differentiation, 4 × 10^5^ cells were pelleted into a micromass by centrifugation at 150 × g for 5 minutes in a 15 ml conical polypropylene tube and cultured in Chondrogenic Differentiation Medium Kit (Cyagen, Silicon Valley, CA, USA) for 3 weeks. The chondrocytes were fixed with 4% paraformaldehyde, then paraffin-embedded and finally stained with Alcian blue solution.

**Culture of *Helicobacter pylori***

The *H. pylori* strain SS1 was cultured at 37°C under microaerophilic conditions in liquid *H. pylori* Medium (HB8647, Hope Bio-Technology, Qingdao, China) supplemented with 7% FBS (Gibco) and a standard Campylobacter selective supplement (HB8646a, Hope Bio-Technology, Qingdao, China). To collect the *H. pylori* supernatant, *H. pylori* was cultured in liquid medium for 24h and centrifuged (4000 RPM, 15min). Then the supernatant was filtered by 0.22um filters (Millipore, Merck KGaA, Darmstadt, Germany) and stored at -80℃. *H. pylori* concentration was estimated by measuring the OD600nm, where OD600nm corresponds to ~2 ×10^8^ CFU/mL as previously reported. The ultrasonic crushing of *H. pylori* was prepared as previously described^23^. The supernatant of *H. pylori* was added to BM-MSCs at a multiplicity of infection (MOI) of 0-100. The ultrasonic crushing of *H. pylori* was added to BM-MSCs at a MOI of 50.

**Micro-PET/CT imaging in *vivo***

18F-FDG PET/CT (TransPET®BioCaliburn® LH, RAYCAN Technology Co., Ltd., Suzhou, China) was used to evaluate the metastasis of subcutaneous transplanted tumors in nude mice. Metastases were evaluated at 2 weeks and 3 weeks after subcutaneous transplantation in nude mice in control, MFC, MFC + BM-MSCs groups. The mice were fasted for 12h the day before the experiment and were anesthetized by inhalation of 2% isoflurane with the Matrix VIP 3000 system (Midmark, USA) and injected with 200 ± 10 Ci ^18^F-FDG through the tail vein. Sixty minutes after injection of the 18F-FDG dose, PET acquisition was performed in static three-dimensional mode at two contiguous bed positions including the perfusion CT scan range, 7 minutes per bed position for a total of 14 minutes. The scanned mice were put into a shielded space for 10 ^18^F half-lives before normal feeding.

**Quantitative real-time polymerase chain reaction (qRT-PCR)**

Total RNA was extracted using the TRIzol reagent according to the instructions of the manufacturer (Takara, Takara Biomedical Technology Co., Ltd., Beijing, China) and reversely transcribed to complementary DNA using PrimeScript^TM^ Reverse Transcriptase Master Mix (Takara). Quantitative real-time polymerase chain reaction (qRT-PCR) was performed by StepOne^TM^ Real-Time PCR system (Applied Biosystems, Thermos Fisher Scientific). All samples were normalized to mouse GAPDH. All gene primer sequences are shown in **Supplementary Table 2**.

**Preparation of total cell extracts and western blot analysis**

For western blot analysis, cells were lysed in radioimmunoprecipitation assay (RIPA) buffer with 1 × proteinase inhibitor (Roche, Mannheim, Germany) and phosphatase inhibitor cocktails (Sigma-Aldrich). After 30 min on ice, cell debris was removed by centrifugation at 12 000g for 15 min. The total protein concentration of cell lysate from each treatment was determined by BCA protein assay kit (Applygen Technologies, Beijing, China). The protein samples were solubilized in sodium dodecyl sulfate polyacrylamide gel electrophoresis (SDS-PAGE) Sample Loading Buffer (5 ×) (Beyotime Biotechnology, Shanghai, China) and equal amounts of protein samples (40ug/lane) were separated by 10% tris-glycine SDS-PAGE (Beyotime). Size- fractionated proteins were then transferred to polyvinylidene fluoride membranes (Millipore). The membranes were then blocked in 8% nonfat dry milk in Tris-buffered saline-Tween (TBS-Tween) (10 mM Tris, 150 mM NaCl, 0.1% Tween-20, pH 7.4) and were probed with primary antibodies [anti-THBS2 (Abcam) or GAPDH (Jackson ImmunoResearch Inc, PA, USA)]. The detection was accomplished by incubation with horseradish peroxidase (HRP)-conjugated goat anti-mouse or goat anti-rabbit IgG secondary antibodies (Antgene Biotech, Wuhan, China) and enhanced chemiluminescence (ECL) by autoradiography using the Pierce™ ECL Western Blotting Substrate (Thermo Scientific).

**CFSE-labeling assay**

The BM-MSCs were prepared as single-cell suspension, and washed twice with PBS to remove the serum. The cells were resuspended with PBS to 5-10×10^6^/mL and incubated with 1uM 5-(and-6)-carboxyfluorescein diacetate succinimidyl ester (CFSE, Ebioscience) at room temperature for 10 min in the dark. Then, stop labeling by adding 4-5 volumes of cold complete media and incubate on ice for 5 minutes as manufactory’s instruction. The cells were washed 3 times with a complete medium and transferred to a standard 12-well culture plate at a density of 1×10^5^ cells /mL. The next day, the cells were cocultured with the supernatant of *H. pylori* for 12h. Finally, the CFSE-labeled cells were collected and subjected to flow cytometry analysis at 488nm. The fluorescence intensity of CFSE is halved with each successive cell division, and cell proliferation could be easily analyzed by separating dot plots with CFSE fluorescence intensity.

**Transwell migration assay**

The BM-MSCs (1×10^4^) were seeded onto 8.0 μm pore polycarbonate membrane inserts (Corning, NY, USA) in 24-well plates with the various supplement configurations. After 18h, carefully removed the medium within the transwell inserts and gently wiped off the cells of the upper chamber with a swab. Then, cells were fixed with 4% paraformaldehyde for 15min, stained with 1% crystal violet for 30min, and five fields of view were randomly selected to count cells under a phase-contrast microscope.

**Fluorescence immunohistochemistry**

Paraffin-embedded sections from mouse stomach or subcutaneous tumor tissues were stained for α-SMA, and/or green fluorescent protein (GFP). The tissues were fixed in 4% paraformaldehyde and embedded in paraffin blocks. Each block was cut into 4 μm-thick section, and deparaffinized with xylene and rehydrated with ethanol. Then the antigen-retrieval was performed in 1 mM citrate buffer (pH 6.0, BOSTER, Wuhan, China) at 95˚C for 2 min before staining. Sections were then washed with 1×PBS and were subsequently blocked in a 10% normal donkey serum (Jackson Immuno Research) at room temperature for 1h. Primary antibodies used in this study include anti-GFP primary antibody (1:1000, ab5450, Abcam), anti-α-SMA primary antibody (1:200, BM0002, BOSTER), anti-pan-CK primary antibody (1:200, ab86734, Abcam), anti-CD45 primary antibody (1:200, ab40763, Abcam) and anti-CD105 primary antibody (1:200, ab231774, Abcam). Alexa Fluor-conjugated Donkey 488/594 (1:200, Jackson Immuno Research) was used as secondary antibodies. After counterstaining with DAPI (10ug/ml, ANT046, AntGene Biotech) and mounted with Fluoromount-G^®^ (ANT071, AntGene Biotech). Stained slides were stored at 4°C in the dark and images were taken using fluorescence microscope (Nikon, Tochigi, Japan).

**Immunohistochemistry for paraffin-embedded sections**

The fixed samples were embedded and sectioned, then conventional dewaxing and hydration were performed as described above. Sections were incubated with 3% H_2_O_2_ at room temperature for 5-10 minutes to eliminate endogenous peroxidase activity, and rinsed. The antigen-retrieval was performed as described above. Sections were then blocked in a 5% bovine serum albumin (AntGene Biotech) at 37℃ for 30min and incubated with Ki-67 primary antibody (1:200, 12202S, CST). Antibody detection was performed by a 30-min incubation with HRP-labelled anti-rabbit/mouse antibodies (SV0004, BOSTER), rinsing then developed by 5-min incubation with 3,3'-diaminobenzidine (AR1022, BOSTER). Slides were then rinsed and counterstained with hematoxylin.

**Hematoxylin-eosin staining and Giemsa staining**

Sections from each paraffin block were stained with hematoxylin-eosin (HE). Samples were deparaffinized with xylene and rehydrated with ethanol, and stained with haematoxylin and eosin (H&E) and Giemsa staining. Slides were then mounted with coverslips. Slices were observed under the light microscope.

**Flow cytometric analysis for the stomach**

Flow cytometry was used to detect the distribution of GFP-labelled BM-MSCs in the stomach in different groups as described above. Mice were sacrificed, and the gastric tissues of the mice were separated and rinsed in 1×PBS for 3 times. Gastric tissues were then cut into 0.5-1.0 cm pieces, and the pieces of stomach were incubated in 5 ml of predigestion solution ( 1×PBS, 5mM EDTA and 1mM DTT) for 30 min at 37℃ under slow rotation (40g) in a thermal incubator in a 50 ml tube. Pass the remaining pieces through a 100 mm cell strainer, and place them into 50 ml tubes with fresh digestion solution (Dissolve 0.05 g of collagenase D (Roche), 0.05 g of DNase I (Sigma) and 0.3 g of dispase II (Roche) in 100 ml of 1×PBS) for 30 min at 37℃. Combine the supernatants from all digestion steps in a fresh 50 ml tube and centrifuge for 10 min at 500g at 20℃and discard the supernatant. Resuspend the pellet in 200ul cold FACS buffer and subjected to flow cytometry analysis at 488nm.

**Supplementary Fig. 1** Representative photomicrographs of BM-MSCs isolation, culture and identification. **(a)** Primary culture after 24h incubation; **(b)** BM-MSCs after 3th passage; **(c)** BM-MSCs after 6th passage; **(d)** Flow cytometric analysis of CD44, CD73, CD105, Sca-1, CD45 and CD11b, respectively; **(e)** BM-MSCs were cultured in osteogenic medium, adipogenic medium and chondrogenic medium and stained with Alizarin red dye, Oil Red O and Alcian Blue to identify differentiation.

**Supplementary Fig. 2**

**(a)** Representative haematoxylin-eosin staining image of *H. pylori* in the gastric tissue from chronic *H. pylori-*infected mice; **(b)** Representative Giemsa staining image of *H. pylori* in the gastric tissue from chronic *H. pylori-*infected mice; (c) GFP+ cells were detected by FACS staining in stomach in SHAM group, BM-MSCs group, HP group, and HP + BM-MSCs group, respectively.

**Supplementary Fig. 3** No obvious metastasis was observed in MFC group and MFC+ BM-MSCs group 2 weeks after injection. Representative PET-CT images of nude mice after injection of MFC alone and MFC co-injected with BM-MSCs in the right armpit at 2 weeks post-transplantation.

**Supplementary Fig. 4** Genetic profile of the gastric biopsy of the female patient with acute myeloid leukemia receiving a bone marrow transplant from a male donor using the short tandem repeat sequence analysis.
